# On the Many Actions of Ouabain: Pro-Cystogenic Effects in Autosomal Dominant Polycystic Kidney Disease

**DOI:** 10.3390/molecules22050729

**Published:** 2017-05-03

**Authors:** Jessica Venugopal, Gustavo Blanco

**Affiliations:** Department of Molecular and Integrative Physiology, and The Kidney Institute, University of Kansas Medical Center, 3901 Rainbow Blvd., Kansas City, KS 66160, USA; jessica.venugopal@gmail.com

**Keywords:** cardenolides, Na,K-ATPase, endogenous ouabain, polycystic kidney disease

## Abstract

Ouabain and other cardenolides are steroidal compounds originally discovered in plants. Cardenolides were first used as poisons, but after finding their beneficial cardiotonic effects, they were rapidly included in the medical pharmacopeia. The use of cardenolides to treat congestive heart failure remained empirical for centuries and only relatively recently, their mechanisms of action became better understood. A breakthrough came with the discovery that ouabain and other cardenolides exist as endogenous compounds that circulate in the bloodstream of mammals. This elevated these compounds to the category of hormones and opened new lines of investigation directed to further study their biological role. Another important discovery was the finding that the effect of ouabain was mediated not only by inhibition of the activity of the Na,K-ATPase (NKA), but by the unexpected role of NKA as a receptor and a signal transducer, which activates a complex cascade of intracellular second messengers in the cell. This broadened the interest for ouabain and showed that it exerts actions that go beyond its cardiotonic effect. It is now clear that ouabain regulates multiple cell functions, including cell proliferation and hypertrophy, apoptosis, cell adhesion, cell migration, and cell metabolism in a cell and tissue type specific manner. This review article focuses on the cardenolide ouabain and discusses its various in vitro and in vivo effects, its role as an endogenous compound, its mechanisms of action, and its potential use as a therapeutic agent; placing especial emphasis on our findings of ouabain as a pro-cystogenic agent in autosomal dominant polycystic kidney disease (ADPKD).

## 1. Ouabain Structure and Overall Activity

Ouabain is a member of a group of substances known as the cardenolides, one of the two families that form the cardiotonic steroid type of compounds (also called cardiac glycosides). The other family are the bufadienolides, which include bufalin and marinobufagenin, produced by the skin of the toad, *Bufo marinus* [1]. Among other important cardenolides are digitalis, found in the foxglove *Digitalis purpurea*, digoxin, found in *Digitalis lanata*, and oleandrin from *Nerium oleander* [2]. This review discusses the biological relevance and mechanisms of action of ouabain in different cells and tissues, with emphasis on its effects in autosomal dominant polycystic kidney disease (ADPKD). For additional information covering other cardenolides, the reader is invited to visit a series of excellent reviews [3,4,5,6,7,8].

Structurally, cardenolides are composed of a steroidal backbone, a five-membered unsaturated lactone ring at C-17; a hydroxyl group at C-14; and a sugar moiety that varies depending on each particular compound [9]. Cardenolides were first found in plants, with ouabain being extracted from the African climbing plant *Strophantus gratus* and the *Acokanthera ouabaio* tree. Ouabain and other cardenolides were initially used as poisons due to their toxic effects; then, they were found to have beneficial effects when used in controlled amounts, and were incorporated as herbal remedies [10,11]. Over two centuries ago, cardenolides (mainly digitalis and digoxin) began to be used in medicine due to their positive inotropic effects for the treatment of congestive heart failure. This conferred these compounds their general designation as “cardiotonics” [12]. Later, it was found that cardenolides were also useful in the treatment of atrial fibrillation because of their positive chronotropic action [13]. For years, cardenolides were used empirically, without a clear understanding of its mechanisms of action. The observed correlation of a raise in intracellular Na^+^ and increased force of contraction in cardiac fibers treated with cardenolides, along with the inhibition of this effect by K^+^, helped to link cardenolides with the Na,K-ATPase (NKA) [14]. Cardenolides were found to inhibit the activity and the ATP dependent transmembrane exchange of intracellular Na^+^ for extracellular K^+^ that NKA catalyzes [15]. Further experiments established that intracellular Ca^2+^ played a key role in the mechanism of action of cardiotonic steroids. It was shown that NKA inhibition in cardiac cells, causes a slight increase in intracellular Na^+^ and reduces the inward force for Na^+^ movement inside the cell. This secondarily raises cell intracellular Ca^2+^ by slowing down the function of the Na/Ca exchanger, NCX. The higher cytoplasmic Ca^2+^ allows the cell sarcoplasmic reticulum to become replenished with this cation, via the function of the sarcoplasmic reticulum Ca-ATPase (SERCA). This additional stored Ca^2+^ can then be readily available and used by the myocardium to produce a stronger contraction and increased cardiac output [16,17,18]. A mechanism, similar to that of the heart, was also observed in mouse smooth muscle vessels, which has important consequences for regulating vascular tone, arterial peripheral resistance and arterial pressure [19].

As will be discussed below, ouabain also contributes to blood pressure regulation by modulating Na^+^ homeostasis via controlling salt reabsorption in the renal tubular epithelium [20,21]. While ouabain (and also other cardenolides) importantly influences the function of the cardiovascular and renal system, more recently it has been shown that it has a wide variety of effects in other tissues. Due to all the actions, the relevance of ouabain go far beyond its role as a cardiotonic compound.

## 2. The Ouabain Target, Na,K-ATPase

The natural target of ouabain is NAK, the ion transporter that creates the transmembrane Na^+^ and K^+^ gradients, which are essential for maintaining volume, pH, and the secondary transport of salt, essential nutrients (glucose, amino acids) and water in the cell [22]. The ionic gradients generated by NKA also contribute to maintain the resting membrane potential of most cells and allows the generation of the action potential in excitable cells and tissues [23,24]. NKA is a protein complex, composed of an heterodimer of α and β subunits, which sometimes is accompanied by a third hydrophobic polypeptide, the FXYD subunit [15,25,26], all assembled in a 1:1:1 stoichiometry [27].

The NKA α subunit is the catalytic subunit, which contains the binding sites for Na^+^, K^+^, ATP, and the binding pocket where cardiotonic steroids, such as ouabain, dock [28]. Cardenolides bind to the extracellular side of the α subunit, locking the NKA in the E2 phosphorylated conformation (one of two states that the NAK adopts during its reaction cycle), inhibiting NKA enzymatic and ion transport activity [29]. Because of this, relatively high doses of ouabain have been classically used as a tool to specifically define NKA function [29]. The β subunit is a type II membrane protein which aids in the folding and allows the trafficking and membrane stabilization of the NKA α subunit [30,31,32,33]. In addition, the β subunit serves as an adhesion molecule in several tissues, as for example the nervous system, the renal tubular epithelium, and the lung, where it helps maintain the apical to basal polarity of the cells [34,35,36,37]. The FXYD proteins are accessory polypeptides that are not required for NKA catalytic activity, but they contribute to regulating NKA kinetic properties [38,39,40].

The NAK α and β subunits exist as different molecular isoforms. Four isoforms of the NKA α subunit and three different β polypeptides have been identified in mammalian cells (α1, α2, α3, α4, β1, β2, and β3) [41,42,43,44,45]. Also, the FXYD proteins belong to a family of at least seven different polypeptides [46,47,48]. Different assembly of NKA α and β subunits results in the formation of distinct NKA isozymes, which have different functional properties and are expressed in a tissue specific manner [49,50,51,52]. The α polypeptide gives NKA its main kinetic properties and is responsible for the response of NAK to ouabain. NAK isoforms exhibit unique functional properties, with the affinity for ouabain being one of the characteristics that differs the most among the isoforms. This is mainly seen in rodents, in which α1 is exceptionally resistant to ouabain, compared to α2, α3, and α4 that have a progressively increased ouabain affinity [53]. Studies in heterologous expression systems and in genetically modified animals have helped to decipher the functional difference of each NKA isozyme [45].

## 3. Ouabain Actions in Different Cells and Tissues

Ouabain exerts many different effects that are cell and tissue type specific. In general, these effects are achieved at concentrations that are relatively low and close to those reported to be physiological (in the nanomolar range). In contrast, higher ouabain amounts are toxic, leading in most cases to cell death [54,55]. Among the effects of relatively low ouabain concentrations, original studies showed that ouabain can promote growth of myocardiocytes [56,57]. Moreover, in the whole heart, ouabain induces cardiac remodeling and has beneficial effects in ischemia/reperfusion injury in what is called ouabain preconditioning [58,59,60]. Ouabain dependent cell proliferation has also been found in other cell types, including human umbilical vein endothelial cells (HUVEC) [61], bovine, canine, and rat vascular smooth muscle cells [62,63,64]. In rat mesenteric small arteries, maintained in vitro, ouabain regulates intercellular communication, reducing norepinephrine induced vasomotion and desynchronizing Ca^2+^ transients in the cells [65]. These vascular actions of ouabain represent one of the factors that contribute to the hypertensive actions of this cardenolide.

In kidney cells, ouabain enhances the growth of opossum kidney tubular cells and of freshly dissected rat renal proximal tubular cells [66,67]. Ouabain also increased ^3^H-thymidine incorporation and proliferation of renal mesangial cells. Other effects of ouabain in kidney tubular cells include the regulation of sodium reabsorption, modulation of epithelial cell-cell adhesion and attachment, changes in cell communication via regulation of tight and gap junction proteins, control of epithelial ciliogenesis, and regulation of the contractile state and resistance of isolated descending vasa recta [20,68,69,70,71,72,73]. Moreover, ouabain confers protection from the harmful effects that serum deprivation and Shiga toxin have on the kidney [74,75]. In some neuronal cells, ouabain has a trophic effect, increasing the survival of retinal ganglion cells and stimulating the regeneration of retinal interneuronal cells [76,77]. It also favors the growth of cultured rat cerebellar neurons and astrocytes, and augments DNA synthesis and transcription of the proto-oncogenes c-myc and c-fos in pheochromocytoma PC12 cells [78,79]. Other cell types that respond to ouabain by increasing their cell division rate include fibroblasts and Sertoli cells [80,81]. Ouabain has also been suggested to have immunosuppressive effects, based on its capacity to inhibit lymphocyte proliferation, induce apoptosis of human T-lymphocytes, and suppress the mitogen stimulated proliferation of peripheral blood lymphocytes. In addition, ouabain regulates apoptosis, cytokine production and the function of monocytes [82,83,84,85,86,87]. In rat skeletal muscle, ouabain has been shown to control cell metabolism, stimulating glycogen synthesis and reducing glucose oxidation [88]. Altogether, these examples show the variety and complexity of ouabain actions and highlight the important role that this compound plays in the modulation of tissue viability, development and function.

In addition to affecting normal cells, cardiotonic steroids and ouabain also exert a great diversity of effects in cancer cells. These depend on the cancer cell type considered, the species from which the cells are derived, the NKA isoform composition of the cells targeted, and the concentration at which the cardenolide is used. For a full description of the effect of cardiotonic steroids in cancer, several excellent reviews are recommended [89,90,91,92,93,94,95,96]. With respect to ouabain, a complete review of the different responses of neoplastic cells to ouabain is not the intent of this review, however, some examples will be briefly mentioned. Thus, ouabain has been shown to reduce the proliferation of human estrogen-responsive breast cancer cells, but it has variable effects in estrogen independent breast cell lines, favoring growth, activating apoptosis, or inhibiting migration of the cells [97,98,99]. Ouabain inhibits cell growth, stimulates cell detachment, induces autophagic death, and diminish migration of some lung cancer cells, but not others [100,101,102,103]. Ouabain promotes apoptosis and anoikis in prostate adenocarcinoma cells in a time and concentration dependent manner [104,105]. High doses of ouabain cause lymphoma cell death [106,107]; however, it stimulates proliferation of some lymphocytic leukemia cells [108]. Ouabain prevents the growth of medulloblastoma and glioblastoma cells and tumors [109,110,111]. Other effects of ouabain on cancer cells include the increase in proliferation of human colorectal Caco-2 cells, reduction of cell viability of adrenocortical cells, promotion of cell cycle arrest and apoptosis of liver HepG2 cells, and the decrease in growth of human pancreatic cells xenografted in nude mice [112,113,114].

Altogether, these examples suggest an interesting role for ouabain as an anticancer agent for certain neoplastic conditions. However, the potential toxicity and variability of effects of this drug, which also extends to normal cells, imposes important challenges for the use of ouabain or other cardenolides as anticancer agents. Derivatives based on the structure of cardenolides have been developed with the idea to improve their therapeutic index and increase the specific cytotoxic effects of these compounds in cancer cells. [115,116,117].

## 4. Ouabain Activation of Cell Signaling

Early observations on the effects of ouabain where related to the ability of this cardenolide to inhibit NKA activity. The discovery that activation of a cascade of intracellular events takes place in cells upon addition of ouabain opened a new chapter in the field of NKA. As if being essential for its classical ion transport properties was not enough, NKA was found to be the receptor and signal transducer of the effects of ouabain in cells. This attracted the interest of many researchers and triggered an intense search to understand the mechanisms of actions and role of ouabain in different cells and tissues [118,119]. In the early 2000s, work in myocardiocytes showed that ouabain was able to activate various intracellular signaling pathways, including the activation of the tyrosine kinase Src. It was later shown that Src is normally maintained in an inactivated state by its association with NKA, and that Src release from NKA triggers a series of downstream protein phosphorylation events [120,121,122]. This regulation of Src activity by NKA has been shown to depend on protein conformational changes in the structure of the NKA α subunit. Thus, ouabain binding and stabilization of the NKA α subunit in the E2 conformation, allows Src to become free and active. In this manner, NKA functions as a receptor that uses the kinase activation of Src to stimulate different pathways in the cell [120]. Pathways downstream of Src that are involved in NKA signaling involve the epidermal growth factor receptor (EGFR) and the mitogen activated protein kinase (MAPK) pathway. Later, other intracellular messengers were shown to become activated and mediate the effects of ouabain in different cell types. These include the NFkappaB, PI3K-AKT, mTOR, protein kinase C pathways; as well as other cell effectors, such as nitric oxide, reactive oxygen species and changes in intracellular Ca^2+^ concentration [56,66,67,70,123,124,125,126,127,128,129,130,131]. Interestingly, a specific NKA subpopulation located in caveolae and not all the NKA expressed on the cell surface, responds to ouabain with activation of signaling cascades [132]. In this manner, NKA serves as the receptor for ouabain, which by assembling to a multiprotein signaling complex (named the NKA signalosome), functions as the transduction apparatus that transmits and amplifies the effects of ouabain in cells [119]. Interestingly, the signaling capacity of NKA appears to depend on its isoform composition. Thus, insect cells expressing different NKA α isoforms respond to ouabain with dissimilar activation of ERK [127]. A more sophisticated approach to test isoform specific NKA signaling of NKA α2 was used by expressing this isoform in porcine renal epithelial cells deficient in NKA α1. This allowed to test NKA α2 without major contamination of other NKA isoforms. In this system, ouabain was unable to stimulate Src activity, nor did it induce ERK phosphorylation, suggesting that the NKA α2 isoform may not serve the same role in ouabain induced signal transduction than NKA α1 [133]. In conclusion, ouabain exerts its actions by mechanisms that involve a complex cascade of intracellular messengers, which appear to be activated by conformational changes of the NKA α subunit, and that are differentially mediated by the various NKA isoforms.

## 5. Ouabain as a Hormone

While ouabain and other cardenolides were first believed to be only the products of plants, it was later suggested that substances with properties similar to those of ouabain, could be circulating in the blood of mammals. This idea evolved from experiments directed to find the mechanisms underlying salt and water regulation by the kidney [134]. Initial reports showed that salt induced volume expansion in rats caused the release of a natriuretic factor, and that this agent could be transmitted by the plasma of those animals. Evidence was later obtained that this agent inhibited NKA activity [135,136]. In addition, an endogenous inhibitor of NKA was also found in brain and hypothalamus extracts [137]. Direct evidence for the nature of the “natriuretic factor” was obtained when ouabain, or a substance closely related to ouabain, was identified by liquid chromatography, followed by mass spectroscopy in human plasma [138]. These endogenous ouabain like compounds had cardiotonic and vasotonic effects [139]. Several other lines of evidence supported the notion that ouabain could be an endogenous factor. Thus, ouabain, or a ouabain-like substance was isolated from normal bovine adrenal glands, adrenal gland tumors, the nervous system, the media in which adrenal and PC12 cells had been growing, an in Dahl salt-sensitive rats injected intraperitoneally with a Na^+^ load [140,141,142,143]. In addition, anti-ouabain antibodies were shown to decrease renal salt excretion in rats [144,145]. While evidence accumulated for the identity of endogenous ouabain with the plant derived ouabain, the capacity of vertebrates to synthesize the plant steroid containing the rhamnose sugar residue has been questioned [146]. While more experimental evidence is needed to address this point; interestingly, the sugar moiety does not appear to be essential in mammals, at least for the effect of ouabain on systolic blood pressure [147].

A series of additional studies showed that ouabain is primarily synthesized in the adrenal glands and adrenalectomy results in a decrease in ouabain plasma levels [20]. Synthesis of ouabain has been mapped to the zona glomerulosa and fasciculata of the adrenal gland cortex. Cholesterol, pregnenolone and progesterone are precursors for the production of ouabain, which is synthesized following pathways that are shared with those of other steroids. These involve the activity of 3β-hydroxysteroid dehydrogenase and cytochrome P450 [148]. While the adrenal glands are the main site of synthesis, ouabain or similar substances are also produced in the brain, which suggested that this compound is also a neuroendocrine hormone [149]. Interestingly, different stimuli; including angiotensin II, ADH and atrial natriuretic peptide regulate the secretion of ouabain-like substances [146].

Some researchers have challenged the authenticity of ouabain as an endogenous compound. A discussion summarizing different opinions in support and against ouabain as the endogenous NKA regulator can be found elsewhere [150]. The scarce amounts of ouabain, the difficulties in determining it in body fluids with a simple assay, and the inability to easily distinguish it from similar endogenous or exogenous compounds have contributed to the uncertainties surrounding this elusive hormone. In addition, our little understanding of endocrine aspects of ouabain have precluded further advancing our understanding of this cardenolide as an endogenous compound. Despite this, it is clear that in mammals, the ouabain binding site in NKA is of biological relevance, that ouabain exerts significant cell effects, and that these effects mimic those elicited by the endogenous compound. Moreover, ouabain exerts a variety of actions that are different from those of other cardenolides, such as digitalis and digoxin, suggesting the specificity of its role [151].

Supporting the endogenous nature of ouabain is the observation that several conditions are associated with an increase in the endogenous levels of this compound. Essential hypertension, chronic salt intake, congestive heart failure, and pre-eclampsia have been shown to present with higher than normal circulating levels of ouabain [20,152,153,154,155]. Elevated endogenous ouabain is an essential effector in the mechanisms that maintain salt-dependent hypertension, both in rodent models of hypertension, as well as in humans [149,156,157,158]. The mechanisms leading to the hypertensive effects of ouabain are related to the myogenic action that ouabain causes in small arterioles, which contributes to augment total peripheral resistance. These are mediated via changes in intracellular calcium and a Src-mediated cascade of reactions that regulates Na^+^ and Ca^2+^ transport in the cells [149]. Also, central effects have been described, by which ouabain in the hypothalamus raises blood pressure via activation of the sympathetic nervous system [149]. In addition, several lines of evidence suggest that ouabain contributes to the regulation of blood pressure also through its effect as a natriuretic agent. This has been shown in mice, expressing NKA α1 and α2 isoform with changes in their ouabain affinity [159] and in LLC-PK1 pig kidney epithelial cells [160,161]. The natriuretic effect of ouabain has a relatively slow onset, is sustained over time, and is enhanced by acute volume expansion or chronic mineralocorticoid treatment [162,163].

In the heart, high ouabain levels contribute to enhance the adverse cardiovascular outcomes that accompany high blood pressure states [164,165,166], stimulating cardiac hypertrophy and dilation, increasing left ventricular mass [167], and causing disorganization of the cystoskeleton [168]. Important evidence for ouabain induced cardiac hypertrophy was found in mice expressing a ouabain sensitive NKA α1 isoform. This mice, which are more susceptible to circulating ouabain, have an increased propensity to develop cardiac hypertrophy, and heart failure from secondary left ventricular pressure overload [169]. In humans, the correlation found between high endogenous ouabain amounts in blood and left ventricular wall thickness, or dilated myocardiopathy [170,171], has led to the idea of using ouabain blood levels as a maker to monitor the progression of cardiomyopathy [167,172]. Different from the effects of relatively high levels of ouabain, low and safe doses of ouabain have been shown to delay cardiac hypertrophy and failure caused by heart pressure overload [173]. High ouabain levels have also been detected in patients with hyperaldosteronism, secondary to adrenal gland cortical adenoma. Importantly, surgical removal of the tumor was able to lower blood pressure and circulating endogenous ouabain levels in these patients [174]. Plasma ouabain-like activity was found to be elevated during gestation, and especially in pre-eclampsia [155]. Evidence for the role of endogenous ouabain during pregnancy is supported by the finding of elevated levels of the cardenolide in mice expressing a ouabain resistant isoform of the NKA α2 isoform, and the hypertensive phenotype developed by mice expressing a ouabain sensitive α2 isoform [175].

In addition, plasma ouabain is markedly elevated in different situations in which the kidneys are affected. For example nephrectomized rats, experimentally induced uremia in animals, and patients with chronic renal failure, who are subjected to dialysis for kidney disease exhibit high endogenous ouabain levels [176,177,178,179]. Conversely, sustained elevation of endogenous ouabain in circulation has been associated with kidney damage, with particular alteration of podocytes, glomerular changes, and proteinuria [179].

Altogether, the experimental evidence reviewed suggests that exacerbated amounts of ouabain have detrimental effects to the body. The alteration in body salt and fluid, along with the vasoconstrictor effects of ouabain, converge to increase blood pressure. It is apparent that the primary adverse consequences of elevated ouabain depend on the effects of this cardenolide in the cardiovascular and renal systems. However, the secondary harmful outcomes of hypertension in other organs, as well as the additional effects of ouabain in tissues different from the heart and kidney could contribute to disease. Additional research is needed to help us better understand how abnormal ouabain levels contribute to the onset or maintenance of hypertension and other pathological states.

## 6. Ouabain and Autosomal Dominant Polycystic Kidney Disease

Our laboratory has been interested in understanding the role that ouabain plays in ADPKD, a cystic disease of the kidney. This interest developed from our original studies comparing the functional properties of the NKA from human normal renal epithelial cells (NHK cells) and epithelial cells of kidneys obtained from patients with ADPKD (ADPKD cells). Our results showed that the kinetic characteristics of the NKA of ADPKD cells toward Na^+^ and K^+^ were similar to those of NHK cells. However, when dose response curves for the inhibition of NKA activity by ouabain were tested, ADPKD cells exhibited a heterogeneous response to ouabain. Approximately 20% of the total enzyme of ADPKD cells have a high sensitivity to ouabain, with an IC_50_ in the nanomolar range. The remaining 80% of NKA activity, displayed the ouabain sensitivity of the normal kidney, with an IC_50_ in the micromolar range. This indicated that ADPKD cells had a higher capacity to bind ouabain than NHK cells, which prompted us to investigate whether ouabain could affect the course of the disease [180].

ADPKD is a disorder primarily characterized by the formation of multiple fluid filled cysts that affect the kidney [181]. ADPKD is the most common monogenetic disease of the kidney, affecting 1:400–1:1000 births worldwide [182,183]. ADPKD cysts have been shown to be present in the kidneys already at birth, relentlessly expanding over the lifetime of the patient at variable growth rates [184,185]. Despite the continued cystic growth, the overall function of the affected kidneys remains relatively normal due to the compensatory increase in glomerular filtration rate by the non-damaged nephrons [186]. Eventually, at late stages of the disease, the continued cyst expansion mechanically compresses the surrounding kidney parenchyma. This, added to the tissue fibrosis and inflammation that normally accompanies ADPKD, leads to progressive deterioration of renal function, and end-stage renal disease (ESRD) [187,188,189,190]. In the United States, ADPKD is responsible for approximately 10% of all cases of ESRD [181,191], and is the fourth leading cause of renal insufficiency, requiring dialysis and kidney transplantation therapy [192,193].

ADPKD is caused by mutations in the *Pkd1* and *Pkd2* genes that encode for polycystin-1 (PC1) and polycystin-2 (PC2) proteins, respectively [194,195]. The *Pkd1* gene is altered in up to 80–85% of ADPKD cases [196], while *Pkd2* accounts for 15–20% of cases [196]. A variety of mutations, deletions and truncations in either polycystin have been described, which generate similar manifestations of the disease due to haploinsufficiency of the *Pkd* gene [197], PC1 and PC2 are membrane bound proteins; PC1 is a glycoprotein of ~450 kDa, composed of a large N-terminal extracellular region that serves as a protein-protein interaction domain, a series of 11 transmembrane-spanning domains, and a C-terminal portion where G proteins can bind [44,155,161]. PC2 is a ~110 kDa protein with 6 transmembrane domains and cytosolic N- and C-terminal regions. PC1 and PC2 have been found to interact with one another [198,199,200,201] and this interaction is necessary for full function of the complex [198,202,203]. The functions of PC1 and PC2 are not precisely known; however, these proteins influence several important aspects of renal biology. Among their roles are the following: (1) serve as a permeable cation channel, to regulate Ca^2+^, Na^+^, and K^+^ ions in the renal cells [198]; (2) function as a macromolecular receptor, responding to mechanical [204], chemical [205,206], and peptide [207] stimuli; (3) form part as a component of adherens junctions [208] and desmosomes [209,210], to maintain the proper architecture of the renal epithelium; and (4) operate as a signaling platform that modulates intracellular pathways, such as focal adhesion and microtubule stability [211,212,213], PI3K/AKT [214], JAK/STAT [215], and WNT/β-catenin [207,216], which control the normal structure and function of the renal tubules [198,199,200,201].

While the genetic basis of ADPKD are well known, the relationship between the alteration in polycystins and the events that result in cystogenesis remain unclear. ADPKD has a multifactorial pathophysiology. ADPKD cells are characterized by being incompletely differentiated and a first event in ADPKD cystogenesis is the abnormal rate of cell proliferation of the renal tubular epithelial cells [217]. This characteristic causes the altered cells to form focal expansions that will eventually pinch off the renal tubules into isolated cysts that will continue to inexorably expand in size. Once the newly formed cysts separate from the renal tubule, they continue growing by not only cell proliferation, but also by a change in the transporting properties of the epithelium, which favors fluid secretion into the cyst over fluid reabsorption [218]. Other processes that accompany cyst growth include increased apoptosis, changes in lateral cell polarity, enhancement of cell migration, defects in the function of the cell primary cilium, remodeling and abnormal deposition of the extracellular matrix proteins, inflammatory changes, and interstitial fibrosis [219,220,221].

A characteristic of ADPKD is the variable degree in cyst growth, even among individual who share the same polycystin mutation. This shows that other factors, besides the genetic trait, influence the progression of the disease. Several agents have been shown to contribute to renal cyst development and expansion. These include compounds that can increase the cell levels of cAMP, such as caffeine, forskolin, vasopressin and catecholamines; and other compounds, including EGF and prostaglandins. Therefore, the presence of circulating factors on the genetic cystic background, plays an important role in the development and progression of the ADPKD renal cysts [222]. Identification of those factors is of high relevance to both understand regulators of cyst growth and for the development of approaches that, by targeting those factors, could modify the course of the disease.

## 7. Pro-Cystogenic Actions of Ouabain in ADPKD

As mentioned above, the NKA of ADPKD cells exhibits an increased affinity for ouabain. This does not depend on misexpression of NKA isoforms in the cells. We found that, similar to NHK cells, ADPKD cells only express the α1 and β1 isoforms of NKA [180]. Alternatively, the change in NKA ouabain affinity may depend on association with other proteins. Interestingly, PC1 and PC2 have been found to interact with numerous binding partner proteins [223], including the NKA. Thus, the C terminal portion of PC1 associates with the intracellular domain of NKA located between transmembrane domains 4 and 5 [224]. We confirmed this protein-protein interaction and found that expression of the transmembrane and C-terminal domains of PC-1, together with NKA in insect cells, increased the ouabain affinity of the NKA, reaching a value similar to that found in ADPKD cells (unpublished results). In contrast with our results, a shift in ouabain affinity was not found in Cos cells over expressing PC1 [224]. This disparity in results is unclear and may depend on differences in the expression of other proteins that may differentially favor NKA/PC1 interaction in each cell type. While the mechanisms underlying the abnormal high affinity of ADPKD cells to ouabain remain still unclear, this characteristic may have important consequences for the manner in which ADPKD cells respond to ouabain, making the cells more susceptible to the ouabain levels existing in blood. We found that treatment with ouabain significantly enhanced cell proliferation and cell mitotic index of ADPKD cells [180]. This effect was maximal at nanomolar concentrations of ouabain and higher amounts inhibited cell growth. In contrast, ouabain had little effect on NHK cell proliferation. These results were one of the first reports to demonstrate that ouabain enhances cell proliferation in a hyperplastic disorder and agreed with the notion that ADPKD cells show an increased response to proliferative stimuli [85]. The ouabain-induced proliferation of ADPKD cells was found to depend on the presence of cell caveolae and their disruption via cholesterol depletion with methyl-cyclodextrin abolished the effect. We also identified several components of the intracellular pathway required for ouabain induced ADPKD cell growth. Thus, ouabain activated the kinase Src and induced phosphorylation of the epithelial growth factor receptor (EGFR). Downstream effects of ouabain consisted in the increase in activity of B-Raf, phosphorylation of the mitogen-activated protein kinase (MEK) and the extracellularly regulated kinase (ERK). Associated with this cascade of events was the decrease in expression of the cyclin kinase inhibitors, p21 and p27, which normally suppress the G1-to-M transition of the cell cycle [225]. This downstream effect may therefore account for the increase of cell proliferation that ouabain has in ADPKD cells. Supporting these results, stable expression of the C-terminal domain of PC1 in mouse cortical collecting duct cells was associated with an increased ouabain sensitive phenotype and a higher rate of cell proliferation in response to ouabain. These effects required activation of EGFR, Src and MEK [226].

ADPKD is a disease characterized by its slow progression. This has been explained by an imbalance between enhanced cell proliferation and increased rates of cell apoptosis. Evidence for programmed cell death in ADPKD cells have been reported in kidneys from animal models of ADPKD and in kidneys from humans carrying the disease [227,228,229,230]. Furthermore, cystogenesis has been found to be attenuated when apoptosis is inhibited pharmacologically [231]. In addition, the experimental decrease in the expression of either polycystin results in an increased sensitivity to apoptosis [214,232,233,234,235]. We found that physiological levels of ouabain promote a small but significant increase in programmed cell death in ADPKD, but not NHK, cells [236]. This effect also occurred when cell growth was blocked with thymidine, suggesting that the increase in ouabain dependent apoptosis was not secondary to the stimulatory actions of ouabain on cell proliferation. Ouabain affected the expression of the BCL family of proteins, reducing the anti-apoptotic mediator BCL-2 and increasing the pro-apoptotic inducer BAX. In addition, ouabain caused the release of cytochrome c from mitochondria and activated caspase-3, but did not affect caspase-8. This shows that ouabain triggers apoptosis in ADPKD cells by stimulating the intrinsic, but not the extrinsic pathway of programmed cell death. The apoptotic effects of ouabain are specific for ADPKD cells and do not take place in NHK cells. It has been proposed that in the ADPKD cystic epithelium, the loss of some cells by apoptosis may stimulate proliferation of the surrounding cells [228]. Thus, a slight but significant increase in programed cell death, promoted by ouabain, could be a mechanism that aids in the progression of ADPKD [237,238]. In conclusion, ouabain is able to induce both proliferation and apoptosis in ADPKD cells; however, activation of cell growth and death are differentially regulated by ouabain, in favor of cell proliferation. It is interesting to propose that ouabain functions as general regulator of cell viability, by modulating the growth and apoptotic rate in cells.

Another essential mechanism for the formation and growth of ADPKD cysts is the secretion of fluid by the renal epithelium [239,240,241,242]. Treatment with only ouabain did not affect the transepithelial fluid secretion carried on by polarized ADPKD cell monolayers grown on permeable supports. However, ouabain significantly enhanced the cAMP-stimulated fluid secretion in ADPKD cells [243]. These results suggest that ouabain acts as a cofactor enhancing the secretory effects of cAMP in ADPKD cystic epithelial cells. In contrast, ouabain did not have a significant effect in cAMP dependent fluid secretion in NHK cells [243]. The enhancement of forskolin-dependent expansion caused by ouabain was confirmed also in ADPKD cell microcysts grown in a collagen matrix and in metanephric organs from *Pkd1^m1Bei^* mice, a mouse model of ADPKD. Therefore, ouabain helps to increase the size of cysts growing in culture or in the environment of the whole kidney tissue. All these effects of ouabain on ADPKD fluid secretion were abrogated by the pharmacological inhibition of several components of the ouabain-mediated signaling cascade, such as EGRF, Src, and MEK [243]. In this manner, it appears that the main pathway activated by ouabain that induces ADPKD cell proliferation also enhances fluid secretion by the cells.

Ouabain enhanced the short-circuit current of the ADPKD cell monolayer to forskolin, consistent with an increase in cAMP dependent Cl^−^ secretion. This current was found to depend on an activation of the cystic fibrosis transmembrane regulator (CFTR), located on the apical side of the cells. This ouabain induced activation of CFTR appears to be due to an increase in the trafficking of CFTR to the plasma membrane and to up-regulation of the expression of the CFTR activator PDZK1. Subsequent studies showed that ouabain could not induce cyst formation in metanephric organs from a double mutant mouse, containing a mutated form of PC1 and lacking CFTR, supporting the role of this transporter as a downstream effector of ouabain induced cAMP fluid secretion [244]. Cl^−^ secretion via the apical CFTR has been found to be the main mechanism leading to fluid secretion in ADKD. In this manner, our results showed that ouabain contributes to enhance cyst growth by impinging on the key system driving ADPK cyst expansion. In addition, ouabain stimulated the internalization of the basolaterally located NKA α subunit and reduced NKA activity in ADPKD cells. This effect was reflected by a slight increase in intracellular Na^+^ in the cells. These results show that ouabain actions are not local, but can extend to distant regions of the cell, including both the apical and basolateral side of the cells. Moreover, activation of the apically driven secretion of fluid via the CFTR and the reduction of Na^+^ (and presumably water) on the basolateral aspect of the cells by ouabain contribute to the typical change in the directional fluid movement of the ADPKD epithelium, which switches from the normal absorptive into a secretory mode.

More recently, we have found that ouabain also contributes to maintain the de-differentiated state of the cystic renal epithelial cells, which is a characteristic of the ADPKD cell phenotype. Thus, ADPKD cells respond to physiological doses of ouabain with decrease in expression of the epithelial marker E-cadherin and increase in expression of the mesenchymal markers N-cadherin, α smooth muscle actin (αSMA) and collagen-I. This effect, which is not seen in NHK cells, agrees with a role for ouabain in inducing a epithelial to mesenchymal transition (EMT) in the ADPKD cells. However, a complete transition of the cells to a mesenchymal phenotype was not observed, since markers, such as vimentin and fibronectin appeared not to be affected. This suggests that ouabain promotes partial EMT changes in the cells (Venugopal J., Mc Dermott J., Sanchez G., Sharma M., Barbosa L., Reif G.A., Wallace D.P., and Blanco G., Exp. Cell Res. in press).

**Figure 1:**
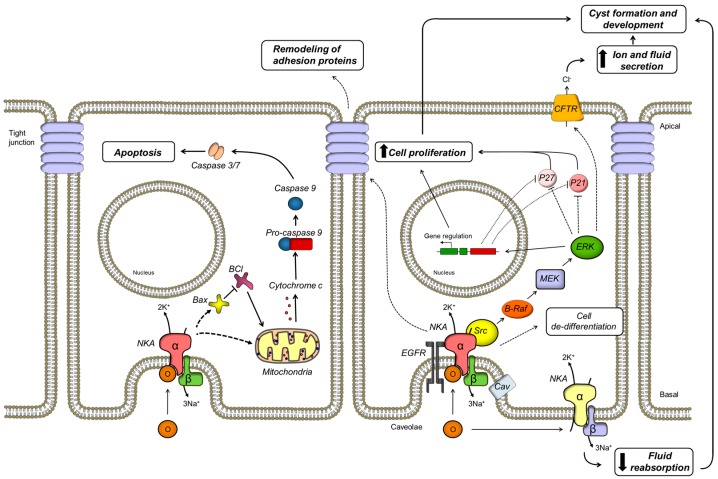
Diagram showing the various effects as well as the different pathways activated by ouabain in ADPKD cells. Ouabain induced and NKA mediated signaling controls ADPKD cell proliferation via the Src-MEK-ERK pathway and cell death via stimulating the intrinsic apoptotic pathway. This effect of ouabain creates a disbalance that favors cell growth, one of the hallmarks of ADPKD cystogenesis. In addition, ouabain stimulates the cAMP dependent secretion of Cl^−^ in the cells by activating the CFTR. This, along with a small reduction in NKA function that lessens Na^+^ reabsorption at the basolateral membrane of the cells, favors epithelial secretion over reabsorption, which helps maintain cyst growth. Also, ouabain induces cell de-differentiation and changes in the expression of adhesion proteins in ADPKD cells, via intracellular pathways that are not yet well characterized. O, ouabain, (α and β) subunits of the Na-K-ATPase. See the text for additional definitions.

Interestingly, ouabain also altered the expression of adhesion molecules in ADPKD cells. Ouabain elevated the expression of the tight junction proteins occludin and claudin-1. However, it did not significantly modified the tight junction protein ZO-1 and the adherens junction proteins β-catenin and vinculin. At the cellular level, ouabain stimulated ADPKD cell migration, reduced cell-cell interaction, and the ability of ADPKD cells to form aggregates. Moreover, ouabain increased the transepithelial electrical resistance of ADPKD cell monolayers, suggesting that the paracellular transport pathway was preserved in the cells. Altogether, these actions favor the ADPKD phenotype, by enhancing the de-differentiated cystic phenotype of the ADPKD epithelium and stimulating cell mobility, which helps ADPKD cells to continue proliferating to increase cyst size. In addition, the remodeling of cell junctional complexes allows the ADPKD epithelium to preserve its structural integrity, which is necessary for the accumulation of fluid within the cysts. These effects further support the key role that ouabain has as a factor that promotes the cystic characteristics of ADPKD cells. A scheme summarizing the various cellular effects and signaling pathways triggered by ouabain in ADPKD are shown in Figure 1.

In conclusion, our findings have identified ouabain as an important novel factor, which by affecting different functions of the ADPKD renal cells, accelerates cyst growth. In addition, studying this disease gave us new insights for the diversity of actions of ouabain on cells. At present there is no data available on the levels of endogenous ouabain in patients with polycystic kidney disease. It is possible that as renal cystic disease progresses to renal insufficiency, the levels of endogenous ouabain may raise, as has been described in chronic renal failure [176,177,178,179]. Then, the pro-cystogenic effects of ouabain will help exacerbate the continuous growth of the cysts. Alternatively, it is also possible that an increase in endogenous ouabain levels may not be necessary to modify the progression of ADPKD. We have shown that ouabain, in concentrations close to those reported to be circulating in plasma of normal individuals, can already stimulate proliferation of the renal cystic epithelium and enhance the cAMP dependent secretion of fluid, due to an abnormally high affinity of the ADPKD cells to ouabain.

ADPKD has eluded successful treatment due to the polymodal and complex nature of its pathophysiology. At present finding therapeutic approaches to treat ADPKD is highly needed to relieve the physical burden of the patients that suffer from this disease as well as to decrease the health care costs associated with palliative measures used to prolong the life of these patients [245]. Although ADPKD is a genetic disorder, its slow progression provides the opportunity to control factors that enhances cystogenesis to delay the progression of the disease. If cystic expansion could be slowed, the destruction of the surrounding renal parenchyma could be decreased, therefore prolonging the functional life of the diseased kidney [246]. Currently, there is no specific treatment for ADPKD approved in the United States, although several guidelines for disease management exist (reviewed in [247,248]). Until genetic tools become available to cure this condition, identification of factors that favor cyst progression provides opportunities to halt or control cyst formation and the advancement and morbidity of the disease. The development of potential pharmacological approaches for ADPKD treatment has been directed toward interfering with the intracellular pathways governing cyst growth [38,139,160,163,190,206,209,215]. Our findings have identified ouabain as an important pro-cystogenic factor in ADPKD. Further studies are underway in our laboratory to continue exploring ouabain effects in ADPKD, as well as approaches to target ouabain induced and NKA medicated signaling that could prove successful in ameliorating the disease.

## 8. Ouabain and NKA Signaling as Targets for the Treatment of Disease

The evidence that high levels of endogenous ouabain play a role in essential hypertension, cardiac hypertrophy and heart failure, stimulated the search for the development of anti-hypertensive agents based on the antagonisms of ouabain’s effect in the cardiovascular system. A digitoxigenin derivative, rostafuroxin or PST 2238, which displaces ouabain binding from NKA was selected for its ability to counteract ouabain effects on renal cells in culture and prevent increased blood pressure and organ hypertrophy in animal models [249,250]. The anti-hypertensive effects of rostafuroxin are due to normalization of the altered function of ouabain induced and NKA mediated signaling and it is not dependent on the associated actions of the compound as a diuretic [251]. While rostafuroxin showed positive effects against specific forms of hypertension (including those associated with increases in endogenous ouabain), and despite the compound was reported to have a high safety ratio in rodent studies, it was not further developed for full approved clinical use [249,252,253].

Another compound that interferes with ouabain action is pNaktide. This is a 20 amino acid long peptide with the sequence of the nucleotide binding domain of the NKA α subunit, which is able to bind to the kinase domain of Src and inhibit its activity [254]. pNaktide was derived from the regulatory function that NKA exerts on Src, via changes in the NKA cycle dependent conformation and its association/dissociation with the kinase. In cultured cells, pNaktide disrupts the formation of the NKA/Src complex, reduces Src function, blocks the ouabain induced activation of Src, phosphorylation of ERK, and the downstream effects of ouabain [255]. Among the actions of pNaktide are blocking the hypertrophic growth of myocytes, proliferation of human prostate cancer cells, and angiogenesis and growth of tumor xenographs [256]. Also, pNaktide attenuates oxidative stress and lipid accumulation in murine pre-adipocytes and reduces body weight gain in mice fed with a high fat diet, suggesting a potential use of the peptide in obesity, insulin resistance and metabolic syndrome [257]. In addition, pNaktide may have protective effects in states in which reactive oxygen species are driving disease. For example, pNaktide, by antagonizing NKA mediated oxidative stress, attenuated the development of uremic cardiomyopathy, a condition in which NKA amplified oxidations are enhanced [258].

An antagonist that has effects in blocking ouabain actions and those of cardenolides in general is Digibind. This is a purified Fab fragment of an anti-digoxin antibody generated in sheep that has been used for the treatment of digoxin intoxication [259]. Consistent with its ability to interfere with cardenolide effects, Digibind has been shown to reduce blood pressure in several rodent models of hypertension when administered in blood or directly in the central nervous system. Also, Digibind reduces natriuresis in normal rats and blocks the effect of cardenolides in blood vessel constriction in rodent models of hypertension and preeclampsia [260,261,262].

## 9. Concluding Remarks

We have come a long way in our journey toward understanding how ouabain and related cardenolides function. Recent advances have revealed unexpected and fascinating aspects of these molecules and through them; we have discovered a new role for its receptor NKA. Once merely recognized for their cardiotonic action, we now know that endogenous and exogenously administered ouabain can exert a myriad of different effects, both in normal and diseased cells. Our unexpected findings of the pro-cystogenic effects of ouabain in ADPKD is another testimony to the multiplicity of actions of ouabain. A better understanding of the properties of ouabain and other cardenolides will allow us to take advantage of these intriguing chemical structures to design new agonist or antagonist agents with improved effectiveness, less toxicity and better therapeutic index. Additional efforts into the development of new compounds, analysis of structure activity, and characterization of selectivity of action toward different NKA isoforms and different cell pathways will be desired future goals in the field of cardiotonic steroid research.
